# Supplementary material for: CellNavi predicts genes directing cellular transitions by learning a gene graph-enhanced cell state manifold
Source: Nat Cell Biol. 2025 Oct 3;27(10):1863–74. doi: 10.1038/s41556-025-01755-1 (PMC12527915; doi:10.1038/s41556-025-01755-1)
Supplement: Supplementary file 2 — Reporting Summary [file 41556_2025_1755_MOESM2_ESM.pdf]

nature portfolio

Corresponding author(s): Pan Deng

Last updated by author(s): Jun 15, 2025

Reporting Summary

Nature Portfolio wishes to improve the reproducibility of the work that we publish. This form provides structure for consistency and transparency in reporting. For further information on Nature Portfolio policies, see our Editorial Policies and the Editorial Policy Checklist.

Statistics

For all statistical analyses, confirm that the following items are present in the figure legend, table legend, main text, or Methods section.

n/a	Confirmed	
☐	☒	The exact sample size (*n*) for each experimental group/condition, given as a discrete number and unit of measurement
☒	☐	A statement on whether measurements were taken from distinct samples or whether the same sample was measured repeatedly
☐	☒	The statistical test(s) used AND whether they are one- or two-sided<br>*Only common tests should be described solely by name; describe more complex techniques in the Methods section.*
☒	☐	A description of all covariates tested
☐	☒	A description of any assumptions or corrections, such as tests of normality and adjustment for multiple comparisons
☐	☒	A full description of the statistical parameters including central tendency (e.g. means) or other basic estimates (e.g. regression coefficient) AND variation (e.g. standard deviation) or associated estimates of uncertainty (e.g. confidence intervals)
☐	☒	For null hypothesis testing, the test statistic (e.g. *F*, *t*, *r*) with confidence intervals, effect sizes, degrees of freedom and *P* value noted<br>*Give P values as exact values whenever suitable.*
☒	☐	For Bayesian analysis, information on the choice of priors and Markov chain Monte Carlo settings
☒	☐	For hierarchical and complex designs, identification of the appropriate level for tests and full reporting of outcomes
☒	☐	Estimates of effect sizes (e.g. Cohen's *d*, Pearson's *r*), indicating how they were calculated

Our web collection on statistics for biologists contains articles on many of the points above.

Software and code

Policy information about availability of computer code

Data collection	All data analyzed within this manuscript are publicly available. No additional software was used for the data collection process.
Data analysis	python==3.8.19, torch==2.4.0, pandas=2.2.2, numpy==1.23.5, scikit-learn==1.5.1, scipy==1.14.1, networkx==3.3, scanpy==1.10.3. cell-gears==0.1.2, pyscenic==0.12.1, renge==0.0.3, cell-gears==0.1.1, goatools==1.4.12, pertpy (2024.04), AutoDock Vina, PyMol. Custom code developed in this study: https://github.com/DLS5-Omics/CellNavi

For manuscripts utilizing custom algorithms or software that are central to the research but not yet described in published literature, software must be made available to editors and reviewers. We strongly encourage code deposition in a community repository (e.g. GitHub). See the Nature Portfolio guidelines for submitting code & software for further information.

Data

Policy information about availability of data

All manuscripts must include a data availability statement. This statement should provide the following information, where applicable:

- Accession codes, unique identifiers, or web links for publicly available datasets
- A description of any restrictions on data availability
- For clinical datasets or third party data, please ensure that the statement adheres to our policy

HCA data for Cell Manifold Model training is downloaded from the HCA data explorer (https://explore.data.humancellatlas.org/projects). The Norman et al., Tian et al. and Srivatsan et al. datasets were downloaded from the scPerturb project on Zenodo (https://zenodo.org/records/10044268). The raw count data of Schmidt et

al. dataset was downloaded from the National Institutes of Health GEO with accession number GSE190604, and its metadata was downloadedd from Zenodo (https://zenodo.org/records/5784651). The Cano-Gamez et al. dataset was downloaded from the Open Target Platform of this project (https://www.opentargets.org/projects/effectorness). The Fernandes et al. dataset was downloaded from ArrayExpress with accession number E-MTAB-9154. The preprocessed Kowalski et al dataset was downloaded was downloaded from Zenodo (https://zenodo.org/records/7619593#.Y-P7Zi1h2X0). The preprocessed Kowalski et al67 dataset was downloaded was downloaded from Zenodo (https://zenodo.org/records/7619593#.Y-P7Zi1h2X0). For trajectory reconstruction, we used the dataset from GSE132188. For scRNA-seq alignment across varying sequencing depths, we used data from GSE84133, specifically the Human3 sample. PDB entry: 3MAX, 5G3W.

Research involving human participants, their data, or biological material

Policy information about studies with human participants or human data. See also policy information about sex, gender (identity/presentation), and sexual orientation and race, ethnicity and racism.

Reporting on sex and gender	N/A
Reporting on race, ethnicity, or other socially relevant groupings	N/A
Population characteristics	N/A
Recruitment	N/A
Ethics oversight	N/A

Note that full information on the approval of the study protocol must also be provided in the manuscript.

Field-specific reporting

Please select the one below that is the best fit for your research. If you are not sure, read the appropriate sections before making your selection.

☒ Life sciences ☐ Behavioural & social sciences ☐ Ecological, evolutionary & environmental sciences

For a reference copy of the document with all sections, see nature.com/documents/nr-reporting-summary-flat.pdf

Life sciences study design

All studies must disclose on these points even when the disclosure is negative.

Sample size

All data used in this study were obtained from publicly available sources, and sample sizes are consistent with those reported in the original publications. No additional selection was performed, except for the data exclusion criteria described below.

References for test datasets:

1. Schmidt, R. et al. CRISPR activation and interference screens decode stimulation responses in primary human T cells. Science 375, eabj4008 (2022).
2. Norman, T. M. et al. Exploring genetic interaction manifolds constructed from rich single-cell phenotypes. Science 365, 786–793 (2019).
3. Cano-Gamez, E. et al. Single-cell transcriptomics identifies an effectorness gradient shaping the response of CD4+ T cells to cytokines. Nat Commun 11, 1801 (2020).
4. Fernandes, H. J. R. et al. Single-Cell Transcriptomics of Parkinson's Disease Human In Vitro Models Reveals Dopamine Neuron-Specific Stress Responses. Cell Reports 33, 108263 (2020).
5. Srivatsan, S. R. et al. Massively multiplex chemical transcriptomics at single-cell resolution. Science 367, 45–51 (2020).
6. Kowalski, M. H. et al. Multiplexed single-cell characterization of alternative polyadenylation regulators. Cell 0, (2024).
7. Baron, M. et al. A Single-Cell Transcriptomic Map of the Human and Mouse Pancreas Reveals Inter- and Intra-cell Population Structure. cels 3, 346-360.e4 (2016).
8. Bastidas-Ponce, A. et al. Comprehensive single cell mRNA profiling reveals a detailed roadmap for pancreatic endocrinogenesis. Development 146, dev173849 (2019).

Data exclusions

For Schmidt dataset, we excluded cells not mentioned in metadata and removed genes appeared in less than 50 cells. For Cano-Gamez et al., clusters 14-17 were excluded as their source cell could hardly be decided. For Kowalski et al., we excluded perturbations which consist of less than 200 cells.

Replication

This is not relevant for our study since we did not perform any wet-lab experiment. The replication of computational experiments were done with cross-validation, by partitioning data into training and testing subsets across multiple folds and evaluate the overall performance of models, when possible (Supplementary Table 4, Supplementary Table 5, Figure 3h), to confirm that model predictions are robust across different data subsets.

Randomization

This is not relevant for our study since we did not perform any wet-lab experiment

Blinding

This is not relevant for our study since we did not perform any wet-lab experiment

Reporting for specific materials, systems and methods

We require information from authors about some types of materials, experimental systems and methods used in many studies. Here, indicate whether each material, system or method listed is relevant to your study. If you are not sure if a list item applies to your research, read the appropriate section before selecting a response.

Materials & experimental systems

n/a	Involved in the study
☒	☐ Antibodies
☒	☐ Eukaryotic cell lines
☒	☐ Palaeontology and archaeology
☒	☐ Animals and other organisms
☒	☐ Clinical data
☒	☐ Dual use research of concern
☒	☐ Plants

Methods

n/a	Involved in the study
☒	☐ ChIP-seq
☒	☐ Flow cytometry
☒	☐ MRI-based neuroimaging

Plants

Seed stocks

Report on the source of all seed stocks or other plant material used. If applicable, state the seed stock centre and catalogue number. If plant specimens were collected from the field, describe the collection location, date and sampling procedures.

Novel plant genotypes

Describe the methods by which all novel plant genotypes were produced. This includes those generated by transgenic approaches, gene editing, chemical/radiation-based mutagenesis and hybridization. For transgenic lines, describe the transformation method, the number of independent lines analyzed and the generation upon which experiments were performed. For gene-edited lines, describe the editor used, the endogenous sequence targeted for editing, the targeting guide RNA sequence (if applicable) and how the editor was applied.

Authentication

Describe any authentication procedures for each seed stock used or novel genotype generated. Describe any experiments used to assess the effect of a mutation and, where applicable, how potential secondary effects (e.g. second site T-DNA insertions, mosiacism, off-target gene editing) were examined.
